# A Comprehensive Review of Milk Components: Recent Developments on Extraction and Analysis Methods

**DOI:** 10.3390/molecules30091994

**Published:** 2025-04-30

**Authors:** Maria Assunta Acquavia, Antonio Villone, Roberto Rubino, Giuliana Bianco

**Affiliations:** 1 Dipartimento di Scienze di Base e Applicate, Università degli Studi della Basilicata, Via dell’Ateneo Lucano, 10, 85100 Potenza, Italy; maria.acquavia@unibas.it (M.A.A.); antonio.villone@studenti.unibas.it (A.V.); 2 Associazione Nazionale Formaggi Sotto il Cielo–Anfosc, Via San Leonardo 62/A, 84131 Salerno, Italy; presidente@anfosc.it

**Keywords:** milk, macro–micro constituents, analytical methods, chromatography, mass spectrometry, UV-Vis

## Abstract

In recent years, there has been a growing interest in identifying both the macro- and micro-constituents of milk, as their functional and health benefits have been recognized. This review provides a comprehensive overview of all milk constituents, including fats, proteins, oligosaccharides and polysaccharides, vitamins, minerals and polyphenols, along with their extraction protocols and the analytical methods employed for their separation and detection over the last ten years. The principles, strengths and limitations of these methods are discussed, highlighting their importance in advancing milk research. Traditional methods such as spectrophotometry, and advanced technologies, like mass spectrometry and omics approaches, are described to collectively enable a comprehensive understanding of milk’s nutritional and functional components. Election criteria for the analytical platforms suitable for target analytes are provided.

## 1. Introduction

Milk, a complex and nutrient-rich biological fluid, serves as a critical source of nourishment for humans and animals. It comprises a wide array of macro- and micro-constituents, including proteins, fats, carbohydrates, vitamins, minerals and bioactive compounds such as polyphenols and peptides [1,2,3]. Efforts to scale up milk production with the aim of reducing reliance on imports and ensuring sustainability are currently being made, even though it presents several complex challenges. Firstly, infrastructure limitations, including cold chains and processing facilities, may be inadequate for handling increased volumes. This can lead to spoilage, contamination and overall inefficiency in distribution systems [4]. Environmental concerns also arise, such as increased water use, land degradation and methane emissions from dairy farming. As alternatives, non-thermal treatment methods like high pressure processing (HPP) and pulsed electric fields (PEF) appear to be more sustainable solutions. They extend shelf life, retain nutrients and improve safety without high heat [5,6]. Moreover, inconsistent results due to variable raw milk composition can be addressed with better pre-treatment controls, and real-time monitoring tools can ensure that treatment efficiency remains consistent. Understanding the composition and quality of milk is essential not only for nutritional research but also for ensuring safety, authenticity and compliance with food regulations [7,8]. This has led to the development and optimization of various analytical techniques to accurately quantify and characterize milk’s constituents.

The analysis of milk involves unique challenges due to its complexity, with components spanning a wide range of molecular sizes, polarities and concentrations. Furthermore, the presence of a complex matrix of proteins, fats and carbohydrates can interfere with the detection and quantification of trace components [9]. Key issues include matrix effects during ionization in mass spectrometry, interferences in spectroscopic measurements and the need for rigorous sample preparation protocols to isolate target analytes [10]. To this regard, pre-concentration and purification techniques such as solid phase extraction (SPE) can help remove interfering matrix components before analysis, while the use of validated and reproducible protocols tailored to specific metabolite classes allows for target analysis. Modern analytical methods must overcome these challenges while providing high sensitivity, specificity and reproducibility. Recent advancements in analytical chemistry have introduced techniques like ambient ionization mass spectrometry (MS), which enables direct and automated analysis of milk with minimal sample preparation [11]. Moreover, omics approaches, including proteomics, lipidomics and metabolomics, are increasingly being integrated with advanced analytics to comprehensively study milk’s constituents and their interactions [12,13,14]. After a brief description of the main classes of macro- and micro-constituents of milk, this review focuses on the analytical protocols employed in the last ten years for their extraction and analysis, highlighting their principles, strengths and limitations. Relevant papers related to the topics under review were searched in internationally available databases, i.e., Scopus, Google Scholar, Web of Science, Science Direct and PubMed. As keywords, three different ones were chosen, namely milk micro-constituents’ analysis, milk macro-constituents’ analysis and milk metabolomics. After an initial screening of titles, abstracts and keywords, eligible articles were downloaded and carefully reviewed to evaluate the relevance of their methodology, results and conclusions. Publications without available full-text and no significant contribution to the analytical field were excluded.

## 2. Milk Macro-Constituents

### 2.1. Lipids

Lipids are one of the most abundant classes of milk constituents. In general, they account for 6% of the total mass content of milk and mainly include triacylglycerides [15]. Indeed, 98% of milk fatty acids are found in the form of triesters of glycerol, while the remaining 2% comprises other glycerol esters (mono- and di-), phospholipids, glycolipids and free fatty acids [16]. About half of the fatty acids occurring in milk have a full saturated chain made of 18, 16 or 14 carbon atoms, i.e., C18:0, C16:0 and C14:0 [17], while unsaturated fatty acids are generally minor components compared to the total.

**Figure 1:**
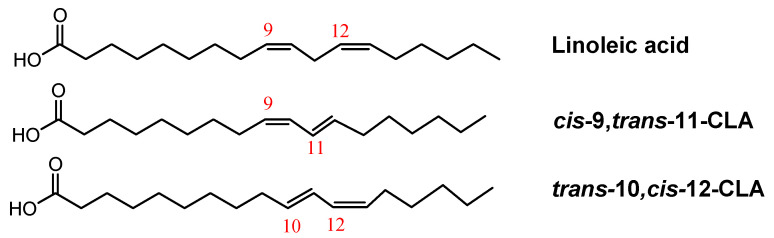
Structure of linoleic acid and examples of conjugated linoleic acids, i.e., *cis*-9, *trans*-11-CLA and *trans*-10, *cis*-12-CLA.

Unsaturated *trans* fatty acids have at least one unsaturation which is in *trans* configuration [18]. A particular class of trans unsaturated milk fatty acids is represented by the conjugated linoleic acids (CLAs). They are a group of geometric and positional isomers of C18 linoleic acid containing conjugated double bonds [19], usually found in positions 8 and 10, or 9 and 11, 10 and 12 and 11 and 13. Since the double bonds are not separated by a methylene group, geometric or *cis-trans* isomers could be distinguished (Figure 1) [20].

Saturated fatty acids and unsaturated *trans* fatty acids are responsible for the decrease of HDL (high-density lipoprotein) values in the blood [21], which is considered one of the main causes of cardiovascular diseases resulting from hypercholesterolemia [22,23].

Another important group of milk lipids is represented by branched-chain fatty acids (BCFAs), which include fatty acids with a methyl group placed on the C2 or C3 carbon, numbering starting from the ω-carbon of the chain. At least 56 isomers of branched fatty acids have been identified in milk, with a carbon chain length ranging from 4 to 26 carbon atoms [24]. Table 1 reports the mean content of BCFAs in fats from bovine milk [25].

### 2.2. Sugars

The main sugar in milk is lactose, an *O*-β-d-galactopyranosyl-(1-4)-d-glucopyranose, which makes up 4.2–5% of the total mass content of milk. Lactose is a disaccharide including α-d-glucose (or β-d-glucose) and β-d-galactose [26]. The C1 atom of glucose can easily change its configuration via mutarotation giving the α and β forms of lactose. The two forms have different specific optical rotations, i.e., +89.4° and +35.0° at 20 °C, respectively. In milk, they are found in a β/α ratio of approximately 1.68. The α-lactose monohydrate is obtained from milk through a process of crystallization at moderate rates and temperatures below 93.5 °C [27]. This method is commonly used in dairy processing and for the production of lactose-based products, such as in the pharmaceutical industry where lactose is often used as a carrier for drugs. Lactose digestion and absorption in the small intestine involves its hydrolysis by the lactase enzyme in glucose and galactose [28]. Hence, lactose represents the principal source of long-term energy in milk [29,30,31]. Recently, the attention to lactose-free products has increased due to the widespread prevalence of lactose intolerance, which results from a deficiency of the lactase enzyme in the jejunum [32].

### 2.3. Proteins

Milk contains a variety of proteins that play essential roles in its nutritional and functional properties [33,34]. Around 200 different proteins have been isolated in milk, with casein being the most abundant [35]. The amino acids that compose them are essentially in the L-α conformation [36]. All the other proteins different from casein are known as whey proteins and include β-lactoglobulin and α-lactalbumin, known for their gelation properties. Measuring the individual whey protein fractions in whey streams is helpful for monitoring and enhancing the effectiveness of whey fractionation processes [37].

## 3. Extraction Methods for Milk Macro-Constituents

Milk macro-constituents can be extracted through different methods characterized by advantages and drawbacks, which make them more or less suitable for the purpose. All the proposed methods are summarized in Table 2, along with advantages and drawbacks. Lipids are typically extracted from milk using Blich [38] and Folch [39] methods, which involve solvent extraction using mixtures of chloroform, methanol and water. Soxhlet extraction is also used, but it is more complex due to the aqueous nature of milk [40,41]. For milk, Soxhlet extraction could provide a high yield of lipids. However, continuous heating at the boiling temperature could lead to lipid oxidation and degradation of heat-liable compounds [42]. Other studies report the use of the single-phase liquid extraction, which is a type of liquid extraction in which the extracted substance forms a single, homogeneous phase with the feed solution (or solid material), as it mixes uniformly with the extraction solvent [43]. Typically, mixtures of chloroform/methanol (2:1) are used for these purposes [43], allowing the efficient extraction of both polar and nonpolar lipids. In order to avoid hazardous problems, other alcohols (ethanol or isopropanol) can be used to replace methanol [44]. Recently, supercritical CO_2_ extraction, supercritical liquid–liquid extraction and accelerated solvent extraction (ASE) have received more attention [45,46,47,48], as they are often based on automated instrumentation, which makes it possible to minimize extraction times and chemical consumption. Since triglycerides with short-chain are, in general, more volatile than long-chain molecules, they are more soluble in pressurized carbon dioxide at low pressure. With regard to ASE, different solvents have been proposed mainly for the extraction of fatty acids and polar lipids from milk-based products (cheese and mozzarella), such as azeotropic mixtures of cyclohexane and ethyl acetate [47], petroleum ether [48], or mixture of tert-butyl methyl ether, cyclohexane and isopropanol [46].

Another example of a newly introduced technique for lipid extraction from milk is the dispersive micro-solid phase extraction (d-μ-SPE). d-μ-SPE involves the dispersion of a small amount of solid sorbent directly into the liquid sample to adsorb specific analytes. Then, after enough contact time, the mixture is centrifuged to separate the sorbent (with the adsorbed analyte) from the liquid. To retrieve the analyte, a small amount of solvent is added to the sorbent pellet, releasing the analyte into the elution solvent, which then can be analysed by chromatographic techniques like HPLC and GC coupled to different detectors, such as mass spectrometer. For the extraction of milk lipids, mainly C18 and zirconia-coated silica gel have been used as a sorbent [49,50]. The extracted lipids are almost triglycerides, while free fatty acids are typically the minor components [51,52].

Milk polysaccharides, such as lactose, can be easily extracted through a few steps aimed at removing interfering fats and proteins. Lipid interferents are removed by centrifuging milk and collecting the aqueous (non-fat) portion. Then, proteins such as casein and whey proteins, are precipitated through the addition of acetonitrile and removed to minimize contamination in the final polysaccharidic extract. Once proteins and fats are removed, the remaining aqueous solution is treated with alcohols (ethanol or isopropanol) to isolate polysaccharides [51]. Other research reports the use of molecular imprinted polymers (MIPs) for the extraction of milk polysaccharides. MIPs are synthetic polymers engineered to recognize and bind specific molecules, such as lactose, in complex mixtures. For lactose extraction from milk, MIPs offer a powerful and selective approach by mimicking the “lock-and-key” mechanism, allowing to selectively target and isolate lactose even in mixtures containing various other components like proteins, fats and other sugars [52]. In addition, also water extraction is suggested as a green extraction method [53] for milk sugars. Some other green solvents used in the extraction of polysaccharides are ionic liquids based on organic cations, such as imidazolium, pyridinium, pyrrolidinium and organic or inorganic anions such as chloride, dicyanamide and trifluoroacetate [54]. On the other hand, solid phase extraction (SPE) is often applied to extract polysaccharides from milk, while concentrating and purifying them. Typically, cartridges packed using octadecyl silica gel, i.e., reversed phase (C18), are used. Common solvents for conditioning, washing and elution steps include methanol, acetonitrile, water and buffer solutions [55].

As regards proteins, they are extracted from milk mainly with enzymatic methods [56]. These methods rely on the use of specific enzymes to modify, separate, or concentrate proteins and offer a gentle and highly selective approach, often preserving the structure and functional properties of proteins better than harsh chemical or physical methods. A pre-treatment step to remove fats and adjust protein concentration is always carried out before enzyme addition [57]. Another innovative method proposed for milk proteins extraction involves the use of nano micelles that have an aqueous core capable of solubilizing polar substances. These nanostructures can capture and isolate proteins due to their high surface area, selective binding capabilities and ability to work under mild conditions that preserve protein structure [58]. Other research proposes microwave assisted extraction (MAE) for milk proteins, as MAE accelerates the extraction process by uniformly and quickly heating the sample, at the same time enhancing protein yield [59]. It is remarkable to consider that after their extraction, proteins can be separated before further analysis by different techniques, such as two-dimensional polyacrylamide gel electrophoresis (2D-PAGE). 2D-PAGE allows proteins separation according to their molecular mass and isoelectric point [60].

**Table 2:** Commonly used methods for milk macro-constituents’ extraction, i.e., lipids, polysaccharides and proteins, with their advantages and drawbacks.

Extraction Method	Advantages	Drawbacks	Macro-Constituents	Used Solvents	Refs.
Liquid extraction	Available standard methods High yield	Laborious multistep process Use of toxic solvents Solvent residues in the extract	Lipids	Chloroform Methanol Water	[38,39]
			Polysaccharides	Ethanol	[51]
				Ionic liquids	[54]
Supercritical CO_2_ and liquid–liquid extraction	Environment-friendly, non-toxic Food quality grade extract Minimum/zero post-extraction processing	High instrumentation cost High energy requirements	Lipids	CO_2_	[45]
Soxhlet extraction	Available standard methods high yield	Time-consuming Use of toxic solvents High extraction temperature	Lipids	Chloroform	[40,41]
Accelerated solvent extraction (ASE)/Microwave assisted extraction (MAE)	Automated and rapid Low consumption of solvents Commercially available instruments	High extraction temperature ASE/MAE Instruments required	Lipids	Cyclohexane/ethyl acetate Petroleum ether, Tert-butyl methyl ether/cyclohexane/isopropanol	[45,46,47,48]
			Proteins	Water	[59]
Solid phase extraction (SPE)	Low consumption of solvents High yield Pre-concentrated and purified extract obtained	High cost Pre-conditioning and cleaning steps necessary for SPE cartridges	Lipids	1% formic acid in methanol	[49]
			Polysaccharides	Water/acetonitrile	[55]

## 4. Analytical Methods for Milk Macro-Constituents

### 4.1. Analysis of Fatty Acids

**Figure 2:**
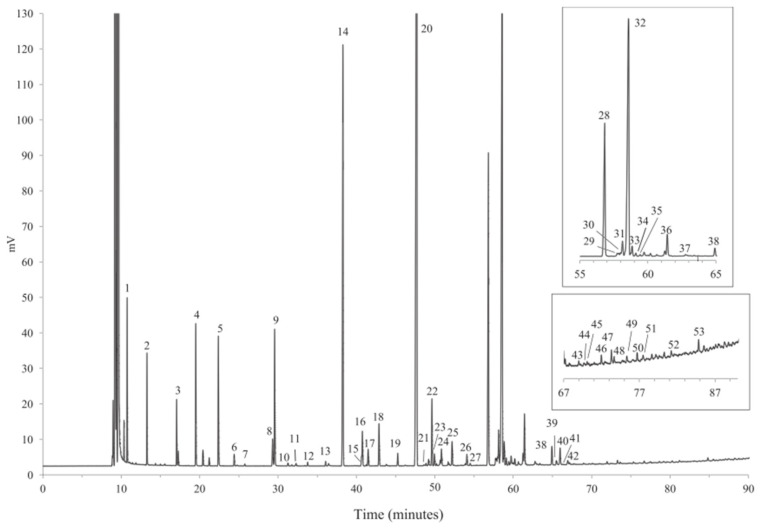
Example of a chromatogram obtained by GC-FID analysis of bovine milk using coating select FAME, 100% bonded cyano-propyl phase, 100 m × 0.25 mm column. Numbered chromatographic signals correspond to identified compounds. Reproduced with the permission of Elsevier [71].

Some of the more conventional techniques that have been extensively used for the analysis of milk fatty acids are gas-chromatography coupled with flame ionization detection (GC-FID) or mass spectrometric detection (GC-MS) [61]. Due to their low volatility and high polarity, both GC-FID and GC-MS methods require a preliminary derivatization step, which involves the esterification of FAs to fatty acid methyl esters (FAMEs). FAs esterification is typically conducted with hydrochloric acid (HCl), acetyl chloride (CH_3_COCl), sulfuric acid (H_2_SO_4_) and boron trifluoride (BF_3_) as derivatizing agents [62,63]. In general, 30 m columns are preferred for the chromatographic separation, as they ensure fast analysis, with good values for limit of detection (LOD) and limit of quantification (LOQ) [64,65]. The dynamic liner range is usually comprised between 10 µg/mL and 500 µg/mL [66], while the LOQ and LOD are around 0.07 µg/mL and 0.02 µg/mL, respectively [67]. Longer columns result in the separation of a higher number of fatty acids [68]. For example, M. Cattani et al. [69] separated saturated, monounsaturated and polyunsaturated lipids with a number of carbon atoms ranging between 8 and 22, with a 40 m column. On the other hand, O. Tzamaloukas et al. [70] used a 50 m column for the successful separation of the CLA isomers, i.e., *cis*-9, *trans*-11 CLA, *trans*-10, *cis*-12 CLA and *trans*-11 C18:1. Moreover, Nina Firl et al. [71] performed the separation of more than 50 different fatty acids, including short-chain (4:0), very long-chain (23:0), highly unsaturated fatty acids (22:5n-3), as well as various branched-chain fatty acids, 18:1 isomers and CLAs, in a 90-min run with a coating select FAME, 100% bonded cyano-propyl phase, 100 m × 0.25 mm column (Figure 2).

**Figure 3:**
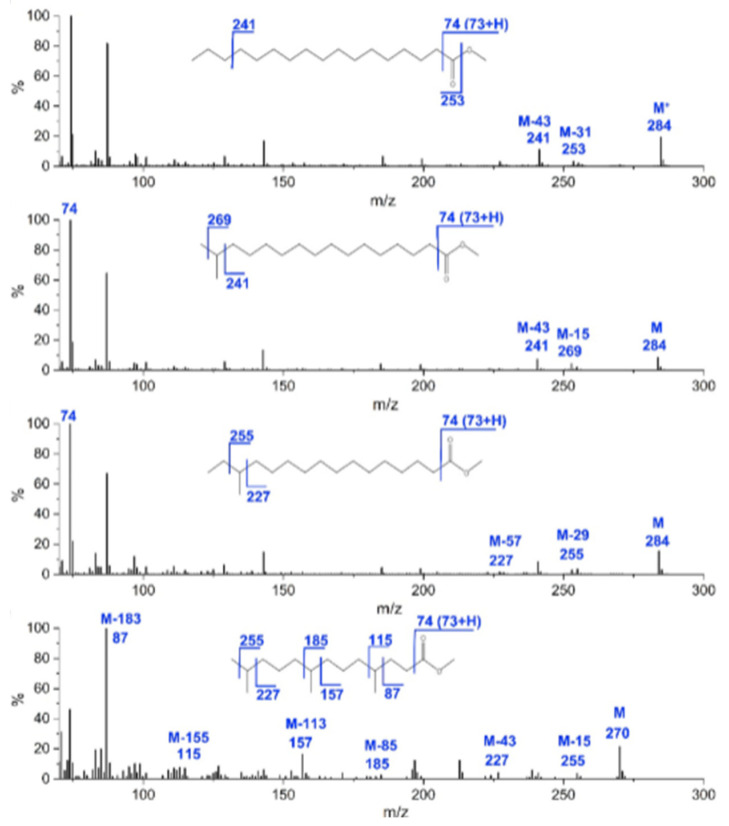
Examples of fragmentation patterns obtained by GC-TOF-MS analysis of FAMEs in yak ghee, i.e., 17:0, i17:0, ai17:0, 4,8,12-trimethyltridecyl acid methyl esters. Reproduced with the permission of Elsevier [73].

Compared to GC-FID, GC-MS has the advantage of providing more structural information, as the comparison with the MS spectral database allows for unambiguous identification of the compounds, while ensuring higher efficiency and better selectivity [72,73]. Electron ionization (EI) is used with different type of MS analyzers, i.e., single quadrupole (Q), ion trap, time-of-flight (TOF). Methyl esters of straight-chain fatty acids have characteristic EI-MS spectra. In particular, [M − 43]^+^ and [M − 31]^+^ ions are characteristic of straight-chain saturated fatty acid methyl esters (n-SFA) (Figure 3). The MS spectra of shorter-chain fatty acids methyl esters typically show a fragment ion at *m*/*z* 74 ([CH_3_–O–C(OH)=CH_2_]^+^) due to γ-hydrogen migration and McDonald’s rearrangement (Figure 3) [73]. Moreover, fragment ions due to McLafferty rearrangement allow the identification of most ester derivatives of fatty acids. With the McLafferty rearrangement, a hydrogen atom from position 4 of the aliphatic chain migrates to the carbo-methoxy group, presumably through a six-membered transition state, which is sterically favored. One result is the elimination of an olefin. If one of the hydrogen atoms on the C4 atom is substituted, then the McLafferty ion will be appreciably lower in intensity than expected [73].

On the other hand, MS spectra of methyl esters of branched-chain fatty acids (iBCFA) show an ion of [M − 43]^+^ corresponding to the terminal isopropyl moiety of the molecules (Figure 3). As regards validation methods, LOD and LOQ values in the range of 9–160 µg/mL and 30–300 µg/mL are typically obtained, respectively [74].

Apart from gas-chromatography, liquid-chromatography coupled to mass spectrometry (LC-MS) has gained a fast development for milk lipidomic applications [75] LC-MS offers several advantages for milk lipid analysis due to its compatibility with a broader range of lipids, soft ionization methods, faster preparation, higher sensitivity and more precise quantification (Table 3). Indeed, LC is often paired with electrospray ionization (ESI) or atmospheric pressure chemical ionization (APCI), which are soft ionization techniques, allowing for the detection of intact lipids and preserving complex molecular structures, like those of glycerolipids and phospholipids. Moreover, sample preparation is easier compared to GC-MS, because lipids can be directly analyzed in their native forms without derivatization. In this way, a great reduction in preparation time and minimization of the risk of chemical alteration is achieved [76]. For the LC separation of the various lipid classes occurring in milk, including triglycerides, phospholipids, sphingolipids and free fatty acids, the choice of the chromatographic column is crucial. Reversed phase columns, mainly C18 or C8 columns, are well-suited for separating nonpolar and moderately polar lipids, i.e., triglycerides, diglycerides and free fatty acids [77]. On the other hand, hydrophilic interaction liquid chromatographic (HILIC) columns are used for the analysis of highly polar lipid species, such as phospholipids, lysophospholipids and gangliosides [78].

LC-MS provides a powerful platform for the comprehensive analysis of milk lipids. The integration of chromatographic separation with mass spectrometry allows for detailed profiling of lipid classes and their structural components, facilitating a deeper understanding of the lipidome in milk. The first stage of mass spectrometry (MS1) provides the molecular ion peaks, which are used to determine the molecular mass of lipid species. Then, tandem mass spectrometry allows to confirm structures by fragmenting the precursor ions. Phospholipids typically undergo neutral loss of phosphoric acid (H_3_PO_4_) or fatty acid residues during MS/MS analysis. For example, in the analysis of PC species, the neutral loss of *m*/*z* 184.0733 corresponds to the choline headgroup, aiding in the identification of PC species [82]. On the other hand, sphingolipids and glycosphingolipids exhibit other characteristic fragmentation patterns. For sphingolipids, the sphingoid base and fatty acid chains fragments are observed in MS/MS spectra. Glycosphingolipids undergo fragmentation to yield ions corresponding to the sugar moieties and the ceramide backbone. For example, in the analysis of lactosylceramide, fragments corresponding to the lactosyl group and ceramide backbone are identified, aiding in the structural elucidation of GSLs [83]. As regards triacylglycerols, they exhibit fragmentation patterns that allow for the determination of fatty acid composition and regiospecific positions. In the analysis of human milk TAGs, MS/MS fragmentation patterns are studied to identify the fatty acid composition and regiospecific positions on the glycerol backbone [83]. Advanced software tools and databases are employed to assist in lipid identification and quantification. Given the structural complexity and diversity of lipid species, these tools are essential for accurate data processing and interpretation. Software such as MS-DIAL (https://systemsomicslab.github.io/compms/msdial/main.html, (accessed on 26 April 2025)) [84], facilitate peak detection, alignment, lipid annotation and quantification using high-resolution LC-MS/MS data. These programs often integrate with in-silico fragmentation libraries and support both untargeted and targeted lipidomics workflows. Complementing these tools are comprehensive lipid databases like LIPID MAPS [85], which provide reference spectra, structural information and biochemical pathways for thousands of lipid species. Together, these resources enable researchers to decode the complex lipid profile of milk with high sensitivity and precision, supporting insights into nutrition, metabolism and infant health.

### 4.2. Analysis of Polysaccharides

The analysis of polysaccharides in milk, such as complex carbohydrates and oligosaccharides, is essential for understanding its nutritional value and health benefits. Official methods are based on Fourier transform infrared spectroscopy (FTIR). FTIR provides a fingerprint of the functional groups in polysaccharides and can be used to confirm the presence of carbohydrates in milk [86]. High-performance anion-exchange chromatography with pulsed amperometric detection (HPAEC-PAD) [87] and LC coupled to refractive index detection (HPLC-RID) [88] have been extensively used for oligosaccharides analysis in milk. HPLC-RID is particularly suitable for detecting and quantifying carbohydrates and polysaccharides, as no UV-active functional groups are requested. On the other hand, LC-MS can be used to detect oligosaccharides and polysaccharides, providing both qualitative and quantitative data, moreover, allowing trace analysis [87,88,89,90,91,92].

### 4.3. Analysis of Proteins

Milk proteomics is the comprehensive study of the protein composition, structure and function of proteins in milk. This field uses advanced analytical techniques, primarily mass spectrometry, to identify the wide array of proteins in milk from various sources, such as bovine, human, goat and sheep milk. The general workflow for analyzing milk proteins by MS involves some key steps after milk defatting and protein precipitation and separation, i.e., digestion and peptide clean-up. Protein digestion involves denaturation, reduction and alkylation and enzymatic hydrolysis. Typically, denaturation is done using urea or other chaotropic agents to unfold their structure, while for the reduction of disulfide bonds and for the alkylation of free sulfhydryl groups, dithiothreitol and iodoacetamide are used, respectively. Finally, trypsin is used to digest proteins into peptides. Other enzymes like chymotrypsin or serine protease Glu-C can also be employed for complementary digestion [93]. In some cases, before digestion, a quantification study could be requested, in order to have preliminary information about protein concentration. Different kinds of assays are available, like Bradford and Bicinchoninic assays. The Bradford assay is based on the binding of tryptophan, tyrosine and phenylalanine aminoacidic constituents of proteins to the Coomassie Brilliant Blue G-250 colorant. A shift from 465 to 595 nm, due to the linkage of the colorant to the aminoacidic components, is observed, which is proportional to the amount of milk proteins. Instead, with the Bicinchoninic (BCA) assay, the quantification is achieved after the absorbance measurement at 560 nm, due to complex formation between Cu ions and cysteine, tryptophan, tyrosine and phenylalanine, enhanced by the addition of BCA [94]. Many other commercial instruments are available for fast protein analysis in milk and are based on absorbance readings in the MID-IR range (9600 nm) [95]. On the other hand, an interesting study by Siročić et al. [96] combined the use of differential calorimetry (DSC), thermogravimetric analysis, Fourier transform infrared and scanning electron microscopy (SEM) techniques, to identify casein in cow milk. With FTIR, it is possible to identify and quantify the protein content based on characteristic amide bands, while SEM is primarily used for studying the microstructure of protein aggregates or protein interactions with other milk components.

Comparing this work with other work based on LC-MS [97,98], it is immediately clear that LC-MS analysis is more laborious as more steps for sample preparation are needed. On the other hand, LC-MS technique provides highly sensitive and specific results, as it can identify low-abundance proteins. Moreover, it is suitable for detecting protein adulteration or contamination [99].

**Figure 4:**
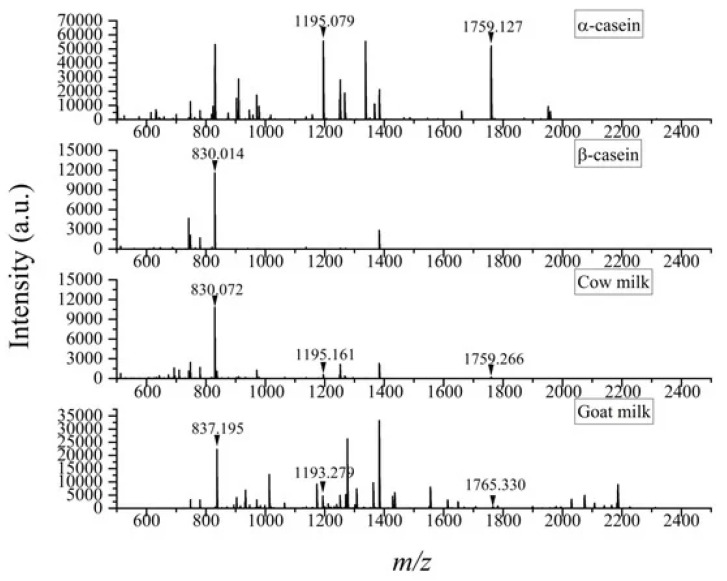
Example of peptide mass spectra obtained by MALDI (+)-TOF MS analysis of α-casein, β-casein, cow milk and goat milk using dihydroxybenzoic acid as matrix. Ions at *m*/*z* 830, 1195 and 1759 used as markers for the identification of cow milk are marked. Reproduced under the terms of the Creative Commons CC BY license [106].

Reverse phase LC has been commonly used to perform the separation of casein in different kinds of milk, including breast milk [93] or to profile milk proteins after heat treatment [100]. As mobile phases, ultrapure water acidified with 0.1% formic acid and acetonitrile are mainly used. After MS or MS/MS detection, the identification of peptide ions for protein sequence analysis is accomplished through the matching of the peptide mass spectra with those available in protein databases like Mascot [101], MaxQuant [102] or Proteome Discoverer [103]. On the other hand, with matrix-assisted laser desorption/ionization mass spectrometry (MALDI-MS) is possible to quickly identify milk proteins or peptides without preliminary extensive separation. MALDI-MS is characterized by simplified workflow and high sensitivity as it allows for the detection of intact proteins or peptide fingerprints in combination with enzymatic digestion [99]. Recent applications of this technique in the field of milk analysis regard the identification of milk adulteration with non-declared animal sources (e.g., buffalo or goat milk mixed with cow milk). This aim is achieved through the identification of specific proteins or peptides that can be considered as markers of milk from an established animal source, such as casein variants [104,105]. For example, ions at *m*/*z* 830, 1195 and 1759 have been found to serve as reliable markers to confirm adulteration with cow milk in commercial products. Indeed, ions at *m*/*z* 1195 and 1759 are ions found in the mass spectrum of α-casein, ion at *m*/*z* 830 is found in the mass spectra of β-casein, while none of them is found in goat milk (Figure 4). Recent studies have demonstrated that adulteration with cow milk in goat milk can be detected at levels as low as 1% with MALDI-MS [106].

## 5. Micro-Constituents

Milk micro-constituents include substances such as vitamins, minerals, biogenic amines, organic acids, nucleotides, polyphenols and animal metabolites. Their number corresponds to several hundred molecules [107].

Vitamins are a heterogeneous group of organic substances essential for the growth and maintenance of the organism. Milk is a source of fat-soluble vitamins (A, D, E and K). Vitamin A is the precursor of β-carotene responsible for the yellow color of cow’s milk. The water-soluble vitamins B1, B2, B6, B12, pantothenic acid, niacin, biotin, folic acid and vitamin C are also present [108].

As regards minerals, they constitute only a small part of milk. Calcium, potassium and sodium are the most abundant cations, while phosphate, citrate and chloride, are the most abundant anions [109]. In milk, they are associated with proteins. In particular, calcium, magnesium, phosphate and citrate form colloidal particles with casein [110].

Biogenic amines (BAs) can be found in milk alongside with some fermented foods such as cheese, sausage, fermented vegetable, wine and fish and they are produced by bacterial decarboxylation of the corresponding amino acids [111]. If present in traces, BAs are essential for life as they involve protein production and DNA replication [112]. However, when occurring in high concentrations, they are toxic and could cause diarrhea and tachycardia [113]. Also, nucleotides, i.e., the precursors of nucleic acids, are involved in improving growth performance [114]. The nucleotides present in milk are ribonucleotides and usually have concentrations in the order of µmol/L [115].

### Polyphenols and Metabolites

Polyphenols are a heterogeneous class of aromatic compounds that have two or more alcoholic groups in their structure. They are usually found in fruit and vegetables but, in many cases, they occur also in milk and dairy products. Polyphenols are exclusively synthesized by plants [116], serving in pollination function, or as a response to environmental stress [117]. Polyphenols in milk can originate from various sources, namely animals’ diet or fortification. If animals consume feed containing polyphenol-rich plants (e.g., grasses, clover, or other vegetation with high polyphenol content), traces of these compounds may be found in their milk. On the other side, some milk products are fortified with polyphenols during processing to enhance their health benefits. For example, dairy manufacturers may add plant-derived polyphenols (e.g., from tea, cocoa, or berries) to create functional or enriched dairy products [118,119,120,121]. The exact type of polyphenols found in milk depend greatly on the animal’s origin and any added ingredients. While raw cow’s milk contains negligible polyphenols, fortified or flavored milk can serve as a vehicle for various polyphenolic compounds, enhancing its nutritional profile.

## 6. Micro-Constituents Extraction

The typical methods for extracting milk micro-constituents involve a variety of chemical, physical and biochemical techniques, essential in dairy science and nutrition research. Table 4 lists the methods that have been used in the last ten years, which will be summarized in the following lines.

One of the main classical and simple extraction methods that suit most of the micro-constituent classes is solvent extraction. Polar or nonpolar solvents can be used to separate vitamins and carotenoids after proteins denaturation [122,123,124]. For bovine milk, a proposed protocol for the isolation of D3 vitamins involves the extraction with hexane/butylated hydroxytoluene [125]. Instead, nucleotides are extracted with water as a solvent, under reflux [126]. The same solvent can be used also for minerals extraction. In this case, a preliminary acid digestion under high pressure is requested [127,128].

Nevertheless, the most modern protocols demand solvent extraction to be assisted by the use of ultrasound or microwaves [128,129,130]. These advanced methods, i.e., ultrasound assisted extraction (UAE) and microwaves assisted extraction (MAE), use energy input (ultrasounds or microwaves, respectively) to enhance the extraction efficiency, speed and selectivity of target compounds from complex matrices and dairy products like milk [131]. Moreover, other works report the use of assisted solvent extraction (ASE), often referred to as pressurized liquid extraction (PLE) for the extraction of fat-soluble vitamins or nucleotides. ASE combines heat and pressure to enhance solvent extraction efficiency, at the same time guaranteeing several unique advantages compared to UAE and MAE, mostly in terms of reproducibility, versatility and compound stability. For vitamins, the extraction is carried out in stainless steel vessels protected by UV-Vis radiation to preserve the compounds oxidation [132,133].

When a further purification and pre-concentration phase is required, solid phase extraction (SPE) is used, thus ensuring a minimal use of sample and chemicals [134]. For example, for BAs, C18, cation exchange, or mixed-mode cartridges can be used. Commonly used solvents to pre-conditionate the cartridges and activate the sorbent are methanol followed by water [135]. To help the release from the milk matrix, biogenic amines previously undergo an acid extraction, often using a solution like 0.1 M HCl [136].

### Extraction of Polyphenols and Their Metabolites

The extraction of polyphenols and their metabolites from milk matrix deserves a separate discussion. As previously stated, when dairy animals consume polyphenol-rich plants (e.g., grasses, herbs and other vegetation), some polyphenols or their metabolites can be transferred into milk. However, this transfer is relatively low because polyphenols undergo significant metabolism in the digestive system of ruminants. As a consequence, their analysis requires accurate and sensitive protocols able to provide the most reliable results, starting from sample preparation and extraction.

Polyphenols often bind with milk proteins through hydrogen bonding, hydrophobic interactions and ionic bonding. Hence, their effective extraction from milk requires separating them from proteins, fats and other matrix components.

One recent method proposed a preliminary milk deproteinization through a one-step homogenous liquid–liquid extraction (HLLE), that uses acetonitrile as a denaturant agent and a salt solution as a separation phase [137]. The canonical precipitation of protein with solvent has been extensively studied to find the best combination of solvent (non-toxic and at a lower cost) that ensures complete precipitation, through the support of vortex, centrifugation or ultrasound treatment. Acetonitrile has been found to be the best option while the vortexing time is not influent [138].

As regards milk defatting, in general, it is carried out after defrosting at 4 °C and then centrifuging at 3000 rpm for 30 min [139]. Once proteins and fats have been removed from the matrix, polyphenols can undergo extraction. Conventional extraction techniques (e.g., solvent extraction) are not always efficient for milk, requiring more complex protocols like solid phase extraction (SPE) or liquid–liquid extraction with multiple steps. Common SPE sorbents include divinyl benzene (DVB), C18 (octadecyl-silica) and porous polystyrene/divinylbenzene (PS/DVB). These sorbents provide a high recovery of polyphenols, and the extraction efficiency does not vary according to the number of extractions [140]. Solvents used for polyphenols elution are acetonitrile/water 1:1 [141] or methanol [142].

Liquid–liquid extraction (LLE), instead, is carried out by using polar solvents assisted by salts (sodium chloride or ammonium chloride) [143]. For example, Nalewajko et al. [143] used ethyl acetate (710 µL) and acetonitrile (510 µL), with the addition of 0.1 g of sodium chloride for a miniaturized and more efficient version of LLE, i.e., dispersive liquid–liquid microextraction (DLLME) of gallic acid, epicatechin, epicatechin gallate, epigallocatechin gallate, daidzein, caffeic acid, quercetin, genistein, naringenin, hesperetin and kaempferol from human milk.

Also, due to their sensitivity to light, pH and temperature, which make them prone to degradation during sample preparation and storage, careful handling, including refrigeration, protection from light, and sometimes pH adjustments, are often necessary to preserve compounds’ integrity. Mildly acidic conditions (pH 3–5) increase the solubility of many polyphenols, minimizing interference from other milk components [144,145].

**Table 4:** Commonly used methods for milk micro-constituents’ extraction with their advantages and drawbacks.

Extraction Method	Advantages	Drawbacks	Micro- Constituents	Sed Solvents	Refs.
Solvent Extraction	Standard methods and high yield	Laborious multistep process and solvent residues in the extract	Vitamins	Water/acid for proteins denaturation	[125]
			Minerals	Nitric Acid 65%	[127,128]
			Biogenic amines	Water/acid for proteins denaturation	[136]
			Nucleotides	Water	[126]
			Polyphenols	Acetonitrile/water	[141]
Ultrasonic Assisted Extraction	High performance/high yield	High cost	Vitamins	Water	[131]
Microwave Assisted extraction	Automated	Use of strong mineral acid	Mineral	Strong mineral acid	[128]
	Commercially available technique		Nucleotides	Water	[146]
ASE/PLE		Special ASE Instrument required	Vitamins	Methanol/methanol-isopropanol	[132]
	Low consumption of solvents Commercially available technique		Nucleotides	Methanol	[126]
DLLME	Low consumption of solvents		Polyphenols	0.1% formic acid solution/water	[142,143]

## 7. Micro-Constituents’ Analysis

The analysis of milk micro-constituents (such as vitamins, minerals, trace elements, bioactive compounds and small molecules like polyphenols and their metabolites) requires a variety of analytical methods due to the diversity and complexity of these compounds.

### 7.1. Spectrophotometric Methods

Spectrophotometric methods are used for the quantification of compounds like vitamins (e.g., vitamin A, D, E, K) and certain bioactive compounds like polyphenols. Several colorimetric assays are available for vitamins determinations.

For example, C vitamin (or ascorbic acid, AA) is determined by a spectrophotometric method which involves cupric ions reduction. Specifically, when present, AA reduces the cupric ions Cu^++^ to cuprous ion Cu^+^ that, in presence of cuproine, forms a complex that adsorbs at 545 nm. This protocol is sensible and provides LOD in order of the mg/L [147].

Also, the total polyphenols content (TPC) can be measured by spectrophotometric assay. This is a fast and sensible method and can be applied to more types of matrixes [148,149]. Spectrophotometric methods for polyphenols content cover a wide range of years and matrix [150,151]. As a limitation, these assays can measure only the total content of phenols, losing the determination of a single phenolic compound.

### 7.2. Electrochemical Methods

Different electrochemical methods have been proposed in the literature for the detection of milk micro-constituents. Recently, the electrochemical-based methods have gained growing attention because the available instruments are portable, fast, simple, inexpensive and have low maintenance costs. Moreover, in many cases, they do not require sample pretreatments. For example, Garehbaghi et al. [152] developed an enzyme-based electrochemical biosensor for sensitive determination of lactose in milk samples. Several studies have used electrochemical techniques to determine antioxidant compounds present in milk samples, notably uric acid (UA), which is present at high concentration in milk. Uric acid is also the final byproduct of purine metabolisms, which is found in a larger amount in biological samples like plasma and urine (UA milk amount < UA blood amount < UA urine amount). Another modified electrode was proposed by other authors, based on a polymer of 3,4-ethylenedioxythiophene (PEDOT), to perform the analysis of uric and ascorbic acid with concentrations in order of the µM [153].

In this study, the electrode modification with PEDOT provided high selectivity towards physiological interfering substances and antifouling properties. When detecting analytes in complex real matrices through oxidation, faradic interference and absorption of large surface compounds can occur. These challenges are typically addressed by electropolymerizing thin organic coatings onto the surface of conventional electrodes, such as glassy carbon or platinum [154].

A new sensor was developed to detect the dopamine in milk, with a copolymer-grafted metal-organic framework that ensured high selectivity and sensitivity [155]. Many other applications are reported for the analysis of adulterant and heavy metals [156].

### 7.3. Atomic Absorption Spectroscopy (AAS) and Inductively Coupled Plasma (ICP)

AAS and ICP are powerful tools for the analysis of minerals and trace elements in milk, such as calcium, magnesium, zinc and iron. AAS has been mainly used for calcium determination in milk. To address chemical interferences, particularly from phosphates, lanthanum is typically added to the sample as a releasing agent (matrix modifier). In flame atomic absorption spectroscopy (FAAS), liquid samples are easily introduced into the analyzer as aerosols following a preliminary treatment, which can involve conventional ashing [157] or wet digestion [158]. Although AAS is fast and less expensive than ICP techniques, it allows for the detection of one analyte at a time and does not provide high sensitivity. Instead, inductively coupled plasma optical emission spectroscopy (ICP-OES) [159] and inductively coupled plasma mass spectrometry (ICP-MS) [160] are both highly sensitive and versatile techniques used for the analysis of trace elements in milk. ICP-OES is more robust and can be used for routine analysis ensuring good LOD and LOQ values. However, without derivatization, it is not possible to analyze mercury and arsenic. ICP-MS allows trace analysis of those metals that require derivatization in ICP-OES. On the other hand, high-cost instrumentations are required. Table 5 compares the performances of ICP-OES and ICP-MS in terms of LOD, LOQ and linearity for the analysis of metals in milk.

### 7.4. Chromatographic Techniques

Liquid-chromatography (LC) and gas-chromatography (GC) are the primary chromatographic techniques used for the determination of milk micro-constituents in milk.

GC-MS is often applied for the fingerprinting of milk volatile compounds responsible for milk flavor, including aldehydes, ketones and esters, which are small molecules often responsible for the flavor and odor of milk [163]. Milk volatiles are extracted through solid phase microextraction from samples headspace, by using divinylbenzene/carboxen/polydimethylsiloxane (DVB/CAR/PDMS) 50/30 µm fiber [163].

In a recent study, Chi et al. [164] found that pyridine, nonanal, dodecane, furfural, 1-decene, octanoic acid and 1,3,5,7-cyclooctatetraene could be used as markers and discriminant compounds of milk originating from different geographical regions of China [164]. This study was made by using a divinylbenzene/carboxen/poly-dimethylsiloxane fiber coupled to an E-nose and a E-tongue. The E-nose contained ten metal oxide sensors for characteristics VOCs and the E-tongue had sensors for human taste (umami, richness (aftertaste-umami), astringency, aftertaste-astringency, bitterness, aftertaste-bitterness, sourness and saltiness). With this approach, it was possible to build the entire organoleptic characteristics of milk, thus identifying milk and dairy product origins [165].

Instead, non-volatile compounds are profiled though liquid-chromatography coupled to different detectors. Reverse phase HPLC (RP-HPLC) are used for the chromatographic separation of different kinds of constituents such as vitamins [166], BAs [167] and nucleosides [168]. For example, nucleoside AMP, GMP and IMP are typically analyzed with C18 column in fifteen minutes with a gradient of sodium acetate and methanol [168].

Normal phase HPLC (NP-HPLC) separation is used for vitamins [169] and for oligosaccharides [170,171]. As an efficient evolution of HPLC in terms of size of particles of stationary phase, UHPLC enables faster separations, significantly reducing analysis time (e.g., minutes instead of tens of minutes). Several applications of UHPLC are available for the analysis of vitamins [172], BAs [173], nucleosides [168], oligosaccharides [174], polyphenols [175] and milk metabolites [176].

In addition, ion-exchange HPLC (IEX-HPLC) is particularly useful for carbohydrates separation [177,178].

#### 7.4.1. LC Coupled Detectors

After the chromatographic separation, several systems are used for milk micro-constituents’ detection. UV-Vis detection is commonly used for vitamins and polyphenols. For vitamins analysis, the most used wavelengths are 247, 280, 320 and 450 nm [179] (Table 6) [175].

A fluorescence detector (FLD) is often used for the analysis of specific vitamins. For example, riboflavin can be detected via HPLC-FLD at 420 nm for excitation and 530 nm for emission as reported in the work of Fracassetti et al. [180]. Another example is tocopherol, determined by HPLC-FLD at 290 nm for excitation and 320 nm for emission [181].

Instead, as most of BAs lack chromophores, derivatization is usually needed to reduce their polarity and improve both chromatographic behavior and UV-Vis detectability [182]. Several derivatizing reagents have been used for LC-UV determination of BAs, with dansyl chloride being the preferred one. This derivatizing agent has been used by Spizzirri et al. [183] to detect BAs in commercial milks for infants and young children. Very low concentrations of BAs were found in ready-to-use liquid milk, with concentrations and profiles related to the production processes of the different samples. A thermogenic accumulation of BAs was noticed in samples manufactured with drastic thermal treatments.

As regards mass spectrometric detection coupled to liquid chromatography for the analysis of milk micro-constituents, different ionization methods are available, each suited for specific types of analytes and applications. Among these, electron ionization (EI), chemical ionization (CI), matrix-assisted laser desorption ionization (MALDI) and electrospray ionization (ESI) are widely used. However, in LC-MS applications for milk analysis, ESI is particularly favored due to its compatibility with liquid phase chromatographic outputs and its ability to ionize polar and thermally labile compounds directly from solution [184].

The application of LC-MS to milk micro-constituents represents a significant advancement in food analysis, enabling the characterization of complex biochemical profiles with a level of detail that was previously unattainable. With its ability to differentiate compounds based on both mass and structural properties, MS coupled with LC remains a cornerstone technique for advancing the understanding of milk’s nutritional and functional properties.

Vitamins such as riboflavin and thiamine, as well as polyphenols introduced through cow feed or fortification, can be effectively analyzed using LC-MS systems. LC-MS and LC-MS/MS are increasingly used to determine vitamin levels in biological samples, offering a reliable alternative to traditional immunoassays due to their ability to simultaneously measure multiple vitamins, namely fat-soluble vitamins (A, D, E) and water-soluble B vitamins. B vitamins, though grouped by their common role as coenzymes or coenzyme precursors, are structurally different, with wide-ranging polarities and molecular weights (123–1579 Da). This chemical heterogeneity makes simultaneous analysis challenging. As a result, analyses often focus on individual B vitamins or small subsets rather than the entire group. Both hydrophilic and lipophilic vitamins are generally analyzed through ESI(+), thus generating protonated molecular ions with predictable fragmentation patterns. For example, vitamin B6 produces a characteristic fragment ion at *m*/*z* 134 due to cleavage of its side chain. Vitamin A (retinol) exhibits fragmentation due to cleavage of the isoprenoid chain, yielding fragments at *m*/*z* 83 and *m*/*z* 93. Specific transitions are selected for multiple reaction monitoring (MRM) MS studies. In detail, fragment ions having the highest intensity (quantifier transition) are used to perform quantitative analysis, while the least intense ones are used for identification purposes (qualifier transition). Table 7 summarizes the qualifier and quantifier MRM transitions used for the detection of the main vitamins occurring in milk [185]. K. Jones et al. [186] developed and validated an LC-MS/MS method for the quantification of 25-hydroxyvitamin D (25(OH)D_2_) and vitamin D_2/3_ (ergocalciferol/cholecalciferol) in human milk. This study found significant variability in milk vitamin D content between individuals, with milk fat concentration being a key predictor. While vitamin D_3_ was the most abundant form, the 25(OH)D_3_ accounted for about two-thirds of the total biological activity, highlighting its greater physiological relevance despite lower concentrations. In another study, Shetty et al. [187] quantified the total B vitamins of fresh cow milk. Their results suggested that fresh cow milk contains higher levels of total B vitamins compared to pasteurized milk, allowing to state that pasteurization may reduce heat-sensitive B vitamins. An inverse relationship was observed between vitamins B5 and B2 across different animal milks. It could be noticed that the B5/B2 ratio may serve as a potential marker to distinguish milk types, as it is typically 1 for goat, >1 for cow and <1 for buffalo milk.

Due to its sensitivity and accuracy, LC-MS/MS is the most used method also for nucleotides analysis. LC-MS/MS based methods allow the simultaneous quantitation of different nucleotides, such as cytidine 5′-monophosphate (CMP), uridine 5′-monophosphate (UMP), adenosine 5′-monophosphate (AMP), inosine 5′-monophosphate (IMP) and guanosine 5′-monophosphate (GMP). Xiao et al. [188] separated these nucleotides by using a C18 column and gradient elution with water containing 0.1% (*v/v*) formic acid and acetonitrile, while their detection was accomplished through an MS/MS system consisted of a triple quadrupole analyzer and an ion source equipped with an electrospray ionization operating in negative mode.

In the context of polyphenol analysis, specific mass detectors like Ion Trap, Orbitrap, Time of Flight and Triple Quadrupole are used due to their unique advantages [184,189].

Rocchetti et al. [190] proposed an UHPLC-QTOF-MS untargeted method to exploit the impact of different pasture-based diets on the chemical profile of Sarda sheep milk, with a particular reference to the polyphenolic composition.

On the other hand, a triple quadrupole MS has been successfully used to validate methods for the determination of phenolic compounds in human milk, highlighting its robustness and sensitivity for such applications [143,191]. Also, in a study of Karunanithi et al. [192], a new analytical method based on LC-ESI-QqQ-MS/MS was developed and validated to quantify the amount of melatonin in milk through solid phase extraction. Moreover, triple quadrupole is ideal for routine targeted quantification of known polyphenols in milk, especially for regulatory and quality control purposes.

#### 7.4.2. Untargeted LC-MS Analysis

**Figure 5:**
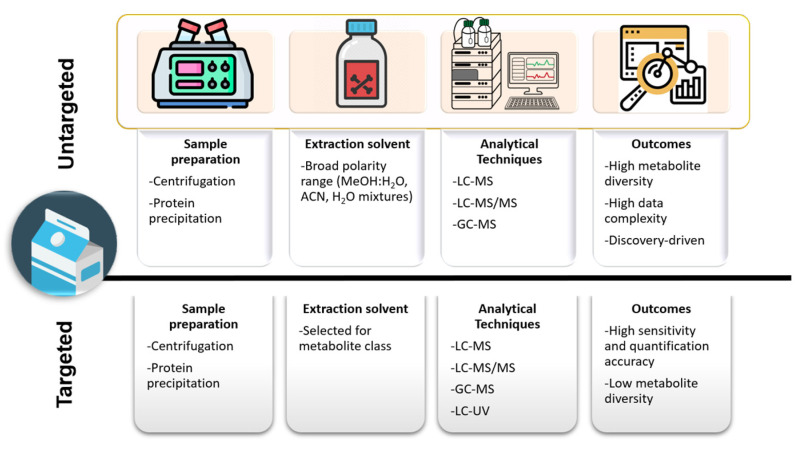
Comparison of untargeted vs. targeted extraction methods for milk metabolite analysis.

Untargeted liquid-chromatography–mass spectrometry (LC-MS) is a powerful approach used to explore the complex chemical composition of milk. Unlike targeted analysis, which focuses on specific known compounds, untargeted LC-MS allows to detect and identify a wide range of molecules without prior knowledge, making it ideal for discovering new bioactive components and understanding the full biochemical profile of milk. The LC-MS generates raw data files for each sample, containing *m*/*z*, intensities and retention times for detected ions. In untargeted workflows, extraction solvents with a broad polarity range are used, to avoid specific metabolite selection (Figure 5). The instrument collects full-scan MS data, sometimes alongside MS/MS data if MS operates in data-dependent (DDA) or independent (DIA) acquisition. The processing of untargeted LC-MS data involves some key steps, i.e., peak detection, deconvolution to separate overlapping peaks and align co-eluting compounds, baseline correction and normalization. Normalization accounts for differences in sample concentration or instrument response. Once compounds annotation has been carried out [193], different statistical tools can be applied to identify significant changes across groups. One of the major aims of untargeted LC-MS analysis is the possibility to study and possibly distinguish between sample types or conditions based on their metabolic or lipidomic profiles. For example, Tane et al. [14] developed a comprehensive untargeted metabolomics strategy based on ultra-performance liquid–chromatography coupled to quadrupole time-of-flight mass spectrometry (UPLC–Q-TOF-MS) to identify potential biomarkers able to distinguish between UHT and reconstituted milk. In another work, Manis et al. [194], evaluated by an untargeted metabolomics approach changes in milk metabolites induced by the replacement of soybean hulls with cocoa husks in the ewes’ diet. Cocoa husks appeared to alter levels of milk metabolites involved in thyroid hormone metabolism and the production of ubiquinol-10.

## 8. Mass Spectrometric Advances in Milk Metabolomic and Lipidomic

Nowadays, the recent introduction of ambient ionization MS techniques has allowed to simplify and greatly shorten the time required for milk metabolomic and lipidomic analysis. Indeed, ambient ionization is a set of ionization techniques used in mass spectrometry that enable the analysis of samples in their natural state with minimal or no sample preparation [195]. These methods work under atmospheric pressure and are designed to ionize molecules directly from complex matrices such as biological tissues, surfaces, or liquids, allowing real-time analysis. Furthermore, great utility comes from the possibility to use smaller, less expensive and potentially portable mass spectrometers, making the technique particularly well suited to in situ or on-site experiments. In the field of milk analysis, ambient ionization is mostly used for authentication studies, to allow the precise differentiation of cow, sheep and goat milks, as well as other dairy matrices. The commonly used techniques are desorption electrospray ionization (DESI) and direct analysis in real-time (DART) coupled to high-resolution mass spectrometry. The primary differences among DESI and DART, lie in their ionization mechanisms and sample handling [196,197,198,199,200]. With DESI, a charged solvent spray is directed onto the sample surface under atmospheric pressure. The interaction between the droplets and the surface causes desorption of analytes, which are then ionized and transferred to the mass spectrometer [198,200]. On the contrary, the mechanism of DART involves the ionization of a stream of heated, inert gas (e.g., helium or nitrogen), thus creating excited-state species [196,197]. These species interact with the analyte in an open environment, transferring energy to produce ions. In this case, the sample is directly exposed to the ionizing gas stream.

In a recent study, Hong et al. [201] developed a DESI-MS based method to detect biomarkers from five distinct animal and plant-based milk sources with the support of chemometric tools. Their method was optimized for MS signal intensity, by choosing a suitable sample treatment. Different protocols were tested, involving milk dilution with organic solvent (methanol) or deionized water. As methanol removed most of the lipids occurring in cow milk, thus causing a loss of biomarkers for milk classification, milk diluted with water in a 1:4 ratio was directly loaded onto the glass slide sample plate and evaporated to dryness at room temperature for DESI-MS analysis. Notable differences in spectral data were observed among cow milk, goat milk, camel milk, soy milk and oat milk. Their results showed that lipid composition can be used as a key factor to discriminate among milk from different species, with sphingolipids (SP) and glycerophospholipids (GP) being more abundant in cow milk. A recent DART-HRMS approach developed by Tata et al. [202] allowed to easy and deep access to the chemical composition of milk directly from samples diluted with an organic solvent. Ethyl acetate was used in this case. The conducted metabolomic analysis demonstrated that monoacylglycerols (MAG) mainly characterize and explain the metabolic variation of grazing milk, while lowland milk is mainly characterized by ketoacid derivates, amines and organic acids.

Hence, the recent advances in the MS field and the introduction of the ambient ionization MS techniques, have revolutionized milk metabolomic and lipidomic analysis by enabling rapid, real-time profiling with minimal sample preparation. These methods allow for precise milk authentication, species differentiation and insights into metabolic variations between grazing and lowland milk, highlighting lipids as key discriminating compounds.

## 9. Conclusions

The diversity of macro- and micro-constituents in milk demands a broad spectrum of analytical techniques, each with unique strengths tailored to specific compounds. From traditional spectroscopic and chromatographic methods to cutting-edge mass spectrometry and omics technologies, these tools collectively enable comprehensive milk analysis. Continued innovations in this field will further enhance the understanding of milk’s nutritional and functional properties, supporting advancements in food science, nutrition and dairy product development. As emerged from the overview discussed in this review, the choice of the analytical platform for milk constituents’ analysis heavily depends on the physicochemical nature and concentration of the target analyte. For instance, macro-constituents such as proteins, fats and carbohydrates are typically present in higher concentrations and are often extracted using liquid–liquid extraction techniques. These methods are straightforward, cost-effective, and do not usually require concentration steps. In contrast, micro-constituents, including vitamins, polyphenols and other bioactive compounds are usually present in trace amounts and require solid–liquid extraction, sometimes combined with pre-concentration techniques (e.g., lyophilization or solid phase extraction) to improve detection limits and reduce sample complexity.

After extraction, spectrophotometric assays are commonly used for general quantification, especially for compounds such as total phenolics or antioxidant capacity. However, because these methods lack specificity, they are often complemented or replaced by selective techniques such as LC-MS/MS or GC-MS, depending on the volatility and polarity of the analyte. For example, GC-MS is preferred for some flavor compounds, while LC-MS is more suited for non-volatile, thermally labile compounds like peptides, vitamins, or phytochemicals.

Furthermore, method development must consider factors such as matrix effects, instrument sensitivity and sample stability. Internal standards and matrix-matched calibration curves are often necessary to achieve reliable quantification. Overall, the use of LC-MS provides enhanced sensitivity and selectivity, enabling the identification of a wide range of milk constituents, even at very low concentrations, making it a powerful tool for both routine quality control and advanced research.

## 10. Perspectives and Challenges

From a comprehensive milk constituent analysis perspective, it is essential for researchers to integrate multiple analytical strategies to maximize the detection and accurate identification of diverse compounds, including proteins, lipids, carbohydrates and metabolites. High-throughput approaches are particularly valuable for initial discovery phases, allowing for the analysis of large sample sets across varied conditions or populations. These broad screenings can then be followed by targeted quantification methods to validate specific biomarkers or functional components. While early studies, especially in milk proteomics, have shown promising results, similar challenges and opportunities exist across other milk components. Developing and refining analytical tools, such as advanced MS-based methods and chromatography, will be key to unlocking insights into milk composition, ultimately supporting advancements in animal health, dairy quality and productivity. 

## Figures and Tables

**Table 1:** Branched-chain fatty acids (BCFAs) composition and content in cow’s milk. The content of FAs is expressed as mg/three daily servings. Reproduced under the terms of the Creative Commons CC BY license [22].

BCFA *	Content (mg/Three Daily Servings)
*iso*-13:0	5–7
*anteiso*-13:0	15–19
*iso*-14:0	18–48
*iso*-15:0	29–97
*anteiso*-15:0	81–206
*iso*-16:0	38–100
*iso*-17:0	58–123
*anteiso*-17:0	24–169
*iso*-18:0	2–20

* *iso*- refers to structures where the branch point is on the second-to-last carbon atom (i.e., one carbon from the end), while *anteiso*- refers to structure where the branch point is on the third-to-last carbon atom (i.e., two carbons from the end).

**Table 3:** Summary of the main advantages and drawbacks of gas-chromatographic and liquid-chromatographic-based methods coupled to mass spectrometry for the analysis of lipids in milk samples.

	Advantages	Drawbacks	Used Chromatographic Columns	Applications	Refs.
GC-MS	Low LOD-LOQ values Profiling of the whole profile of milk fatty acids	Derivatization needed	Bis-cianopropyl-cianopropylphenyl polysiloxane length 30 m to 100 m	Saturated FAMEs from C8:0 to C22:0 and unsaturated isomers	[64,65,68]
LC-MS	No derivatization needed Low LOD-LOQ values	Hight purity and amount of solvents required	HILIC columns C8/C18 columns	glycosphingolipids and gangliosides Free fatty acids CLA isomers	[75,77,79,80,81]

**Table 5:** Comparison of the performances of ICP-OES and ICP-MS in terms of LOD, LOQ and upper limit of calibration range for the analysis of milk trace elements [161,162].

Element	LOD ICP-OES (mg/kg)	LOQ ICP-OES (mg/kg)	Upper Limit of Range ICP-OES (mg/kg)
	LOD ICP-MS (mg/kg)	LOQ ICP-MS (mg/kg)	Upper Limit of Range ICP-MS (mg/kg)
Al	1.08	0.56	125
	0.03	0.1	100
Ca	0.56	1.68	125
	0.89	2.67	1000
Cd	0.18	0.55	125
	0.0001	0.0003	2
Cr	0.06	0.17	125
	0.001	0.003	50
Cu	0.05	0.15	125
	0.012	0.036	200
Fe	0.14	0.46	125
	0.093	0.279	500
Mg	0.05	0.14	125
	0.077	0.231	1000
Mn	0.03	0.1	125
	0.009	0.027	200
Ni	0.67	2.01	125
	0.008	0.024	50
Pb	0.25	0.75	125
	0.003	0.009	5
Zn	0.24	0.72	125
	0.072	0.216	1000

**Table 6:** Wavelength of maximum absorbance for polyphenols and vitamins commonly detected in milk samples. Reproduced with the permission of Elsevier [179].

Analytes	λmax (nm)
Protocatechuic acid	259
(+)-Catechin	278
Gentisic acid	327
Vanillic acid	261
Syringaldehyde	308
p-Coumaric acid	309
Ferulic acid	323
m-Coumaric acid	278
o-Coumaric acid	276
Cinnamic acid	277
Quercetin	372
Kaempferol	366
Retinol (Vitamin A)	323
Vitamin B1	260
Vitamin C	290
Vitamin B7	210
Vitamin B6	210
Vitamin B12	445

**Table 7:** Qualifier and quantifier MRM transitions used for the LC-MS/MS detection of the main vitamins occurring in milk [185,186,187].

	Qualifier Transition (*m*/*z*)	Quantifier Transition (*m*/*z*)
Vitamin A	269/83	269/93
Vitamin B1	265/144	265/122
Vitamin B3	124/78	124/80
Vitamin B5	220/184	220/202
Vitamin B6	170/134	170/152
Vitamin B8	245/123	245/227
Vitamin B9	442/176	442/295
Vitamin B12	678/359	678/147
Vitamin D2	572/298	572/298
Vitamin D3	560/298	560/298
Vitamin E	431/83	431/165

## Data Availability

No new data were created or analyzed in this study. Data sharing is not applicable to this article.
